# Highly Efficient Inverted Light-Emitting Diodes Based on Vertically Aligned CdSe/CdS Nanorod Layers Fabricated by Electrophoretic Deposition

**DOI:** 10.1021/acsami.3c15542

**Published:** 2024-02-15

**Authors:** Yongliang Zhang, Xuan-Manh Pham, Thomas Keating, Na Jia, Anthony Mullen, Devika Laishram, Mei-Yan Gao, Brian Corbett, Pai Liu, Xiao Wei Sun, Tewfik Soulimane, Christophe Silien, Kevin M. Ryan, Zhenhui Ma, Ning Liu

**Affiliations:** † Department of Physics and Bernal Institute, University of Limerick, Castletroy V94 T9PX, Ireland; ‡ Department of Chemical Sciences and Bernal Institute, University of Limerick, Castletroy V94 T9PX, Ireland; § Tyndall National Institute, University College Cork, Cork T12R5CP, Ireland; ∥ Institute of Nanoscience and Applications, Southern University of Science and Technology, Nanshan, Shenzhen, Guangdong 518055, China; ⊥ Shenzhen Key Laboratory of Deep Sub-wavelength Scale Photonics, Southern University of Science and Technology, Nanshan, Shenzhen, Guangdong 518055, China; # Department of Physics, Beijing Technology and Business University, Beijing 100048, China; □ Department of Electrical and Electronic Engineering, Southern University of Science and Technology, Nanshan, Shenzhen, Guangdong 518055, China

**Keywords:** nanocrystal-based light-emitting diodes, inverted architecture, electrophoretic deposition, vertically aligned nanorods, finite element simulation

## Abstract

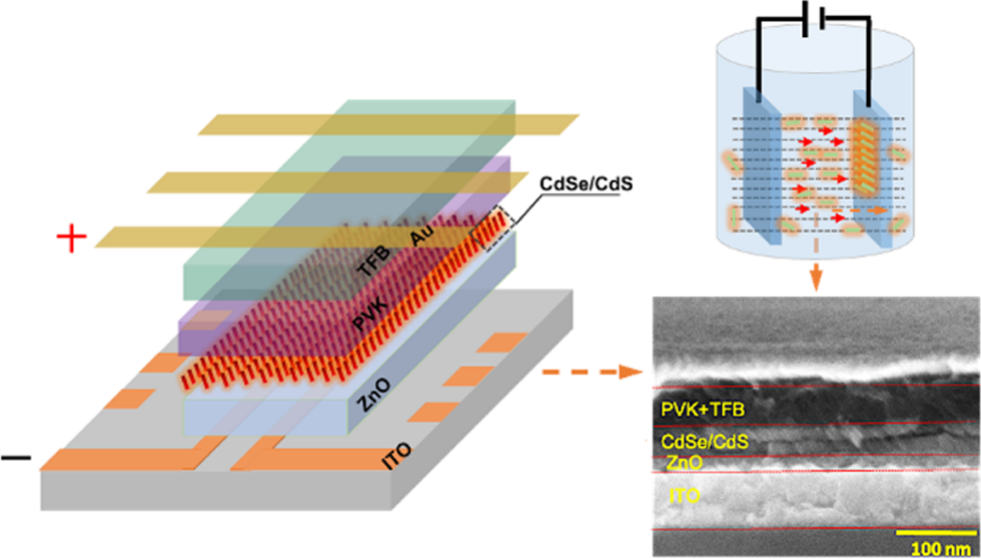

Inverted colloidal-nanocrystal-based LEDs (NC-LEDs) are highly interesting and invaluable for large-scale display technology and flexible electronics. Semiconductor nanorods (NRs), in addition to the tunable wavelengths of the emitted light (achieved, for example, by the variation of the NR diameter or the diameter of core in a core–shell configuration), also exhibit linearly polarized emission, a larger Stokes shift, faster radiative decay, and slower bleaching kinetics than quantum dots (QDs). Despite these advantages, it is difficult to achieve void-free active NR layers using simple spin-coating techniques. Herein, we employ electrophoretic deposition (EPD) to make closely packed, vertically aligned CdSe/CdS core/shell nanorods (NRs) as the emissive layer. Following an inverted architecture, the device fabricated yields an external quantum efficiency (EQE) of 6.3% and a maximum luminance of 4320 cd/m^2^ at 11 V. This good performance can be attributed to the vertically aligned NR layer, enhancing the charge transport by reducing the resistance of carrier passage, which is supported by our finite element simulations. To the best of our knowledge, this is the first time vertically aligned NR layers made by EPD have been reported for the fabrication of NC-LEDs and the device performance is one of the best for inverted red NR-LEDs. The findings presented in this work bring forth a simple and effective technique for making vertically aligned NRs, and the mechanism behind the NR-LED device with enhanced performance using these NRs is illustrated. This technique may prove useful to the development of a vast class of nanocrystal-based optoelectronics, including solar cells and laser devices.

## Introduction

Since the first CdS nanocrystal light-emitting diodes (LEDs) were invented nearly three decades ago,^1^ quantum dot-based light-emitting diodes (QD-LEDs) have been intensively investigated as they have the potential to significantly impact display and lighting technologies due to the unique properties of QDs, such as size-controlled tunable emission wavelength, high quantum yield, and solution processability.^2−5^ In past decades, numerous attempts have been made to improve the device efficiency and luminance by optimizing both materials^6−9^ and device architectures.^2,10,11^ Blue, green, and red QD-LEDs have been fabricated with peak external quantum efficiencies (EQEs) of 21.4, 27.6, and 23.1%, respectively.^12^ Research has shown that a QD-LED device with a few layers of QDs has a high level of luminance and luminous efficiency. Due to the ordered arrays of closely packed QDs, they can minimize the electrical resistance and efficiently enhance the confinement of excitons.^13,14^

Like core–shell QDs, heterostructures based on core–shell semiconductor NRs have a spatial distribution of light-emitting primitives relatively far away from each other, thus effectively suppressing the nonradiative inter-NRs Förster resonant energy transfer (FRET),^15^ and can be used for high-performance NR-LEDs.^16^ Meanwhile, NR heterostructures can provide additional benefits, such as polarized light emission,^17^ larger Stokes shift, faster radiative decay process, and slower bleaching kinetics than spherical QDs.^18^ Therefore, core–shell NRs show good prospects of being applied in optoelectronic devices. Despite these advantages, the deposition methods commonly used in QD-LEDs (such as dip-coating, spin-coating, and spray-cast) typically lead to the formation of a disordered arrangement of NRs that do not maximize the packing density and can also cause gaps or voids that diminish device performance and reliability.

**Figure 1:**
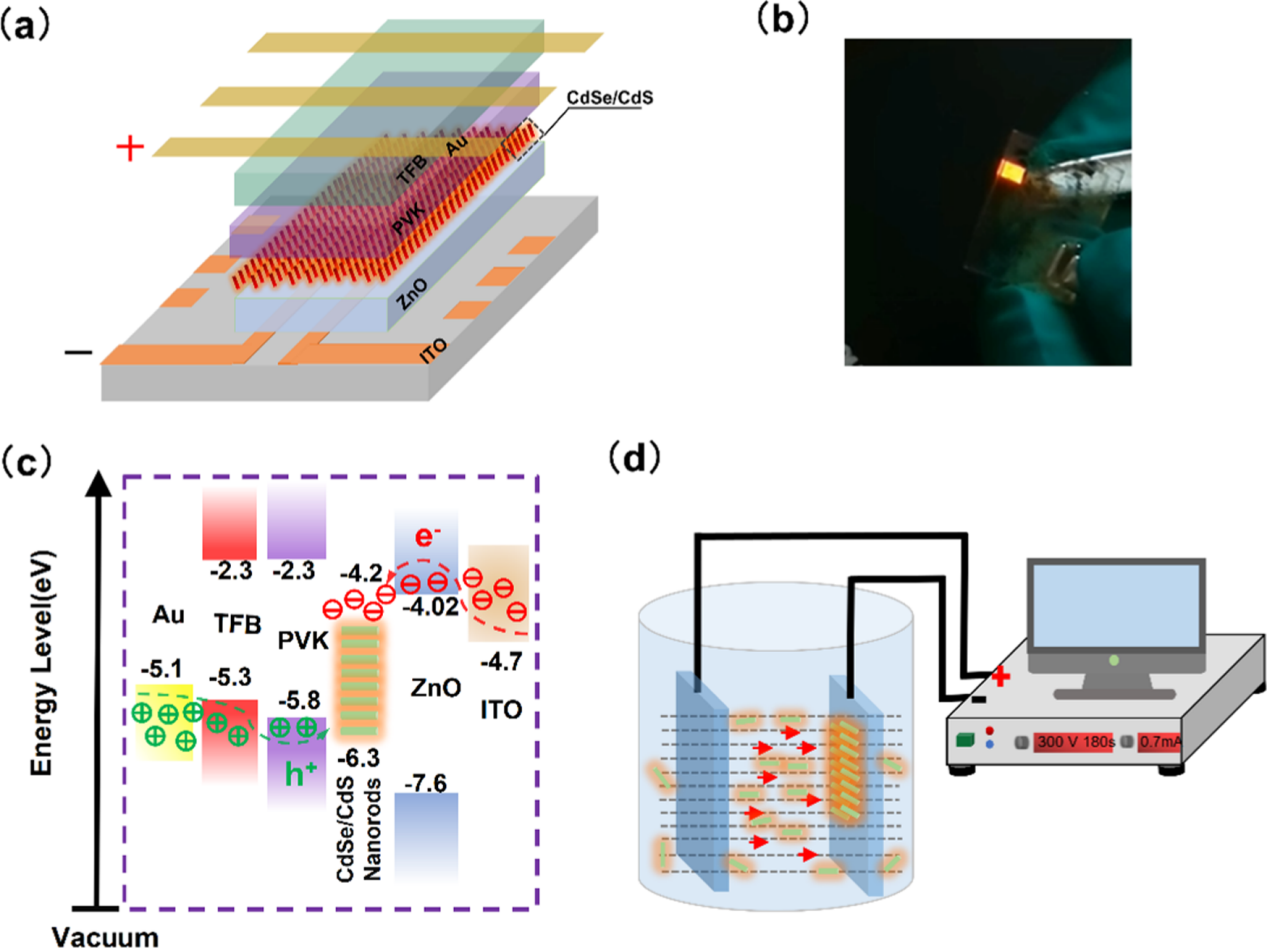
Schematic of device structure and energy levels of NR-LEDs. (a) Device structure. (b) A photo of a working device made by EPD. (c) Energy level alignment for the NR device. (d) EPD schematic diagram.

Different from the spin-coating and spray-casting techniques, this research exploits an electric field-driven technique, namely, electrophoretic deposition (EPD; Figure 1), to form a highly ordered, vertically aligned CdSe/CdS core–shell NR thin film as the emission layer in the device.^19^ EPD allows the CdSe/CdS NRs to be assembled directly from the solution onto conductive substrates. The vertical alignment of NRs takes advantage of the length difference between QDs and NRs; therefore, emissive films with a thickness of over 50 nm can be achieved with only two layers of vertically aligned NRs. As such, this architecture naturally favors directional pathways for charge transfer through the long axis of the NR. In addition, in this work, inverted devices (ITO/electron injection layer/emissive layer/hole injection layer/metal electrode) are fabricated. The inverted structure of an LED can reduce the pixels’ driving voltage and stablize the devices as the cathode of the inverted NC-LEDs can be directly connected to the drain of the thin-film transistor (TFT).^20,21^ Our inverted NR-LEDs demonstrate their best performance with vertically aligned CdSe/CdS NRs as the emitters fabricated by EPD, with a luminance of 4320 cd/m^2^ and an EQE of 6.3%. This is the first time vertically aligned NR layers made by EPD have been reported, and the device performance is one of the best for inverted red NR-LEDs.

## Results and Discussion

The synthesis of TDPA-capped CdSe/CdS core/shell nanorods [transmission electron microscopy (TEM) image in Figure S1a] was carried out according to the seeded-growth approach at high temperatures.^22^ The TEM image of the obtained CdSe/CdS core/shell NRs has a diameter of 4.8 ± 0.2 nm. The mean lateral length and width of the rods were 38 ± 2 nm and 4.8 ± 0.2 nm (Figure S1b), respectively.

As shown in Figure S1c, the upper X-ray diffraction (XRD) spectra show the lattice structure of the CdSe core. The CdSe core has a zinc blende structure compared to the standard CdSe crystal XRD pattern (JCPDF No. 40-0834). The diameter and crystallinity of the CdSe core are calculated by the XRD pattern to be 3.2 nm and 87% (analyzed by JADE software), respectively. The wurtzite crystalline phase and 4.8 nm particle diameter of the CdSe/CdS core/shell NRs were obtained using XRD analysis (Figure S1d). All diffraction peaks can match well with the standard wurtzite CdS crystal XRD pattern (JCPDF of No. 41-1049). The relative crystallinity of the CdSe/CdS NRs is 85.4% calculated by the XRD pattern with JADE software. The diffraction peak of the (002) crystal plane at 26.6° is stronger than the diffraction peaks of other crystal planes, indicating that CdSe/CdS NRs grow along the preferred orientation of the (002) crystal plane. The growth direction [002] indicates that the nanorods are bipolar in nature because of the non-centrosymmetric nature of charge distribution in wurtzite crystals along the *c*-axis. Such a wurtzite structure consists of alternate “Cd^2+^” ions or “S^2–^” planes stacking perpendicular to the length of the nanorod.^23^ This is also the premise and advantage of electrophoretic deposition and will be discussed later.

The optical properties of the CdSe core and CdSe/CdS core/shell nanorods in solution are shown in Figure S1e,f. The CdSe core and CdSe/CdS NRs have an emission of 588 nm with a film photoluminescence quantum yield (PLQY) of 5.3% and 615 nm with a film PLQY of 56.3%, respectively. The full width at half-maximum (FWHM) of the two emission spectra are 27 and 22 nm.

An inverted device structure applied to the NR-LEDs is shown schematically in Figure 1a and consists of the following layers: an indium-tin-oxide (ITO) transparent cathode on a glass substrate, a thin film of ZnO as the electron injection layer (EIL), CdSe/CdS nanorods as the emission layer, a poly(9-vinylcarbazole) (PVK) layer and a poly(9,9-dioctylfluorene-*alt*-*N*-(4-*s*-butylphenyl)-diphenylamine) (TFB) layer as the hole transport layer (HTL) and hole injection layer (HIL), respectively, and a Au anode. The CdSe/CdS core/shell NRs deposited by EPD are the key to the device fabrication. Compared to the spin-coating method, EPD leads to denser films and better adhesion to the substrate. Similar to the sputtering technique, the weakly charged NRs will be accelerated by an applied voltage of 300–500 V. The NRs carry a relatively large kinetic energy (a few tens of eV) once they arrive at the substrate. This ensures that the NRs have good contact with the substrate surface and among themselves. Since the CdSe/CdS NRs are deposited on the ZnO film by EPD in the toluene solution, the solvents used for subsequent HIL and HTL deposition are orthogonal to toluene. The device is fabricated successfully as shown in Figure 1b. The experimental details can be found in the Materials and Methods section.

Figure 1c shows a schematic of the flat-band energy level diagram of the layers. The energy level values for ITO, ZnO, PVK, TFB, and Au were taken from refs (24−26). In particular, as the bottom layer, ZnO deposited by atomic layer deposition (ALD)/e-beam evaporation exhibited excellent stability against various solvents, which is suitable for the CdSe/CdS NR layer deposition by EPD afterward, high electron mobility (∼10^–3^ cm^2^ V^–1^ s^–1^),^27^ and the previously identified benefit of efficient electron injection into the NR layers.^14,28^ Besides, the electron-transporting layer grown by ALD/e-beam evaporation provides more precise control over the layer thickness. The hole transport layer of PVK and TFB takes advantage of the deep highest-occupied-molecular orbital (HOMO) at 5.8 and 5.3 eV, respectively, to realize efficient hole injection and transport into the NR layers.

A schematic of the experimental setup of EPD is shown in Figure 1d. The uniform electric field between the two electrodes is required for the EPD as the electrophoresis can take place in inhomogeneous fields that can significantly affect the deposition process.^29^ The mass deposited, ignoring the charge carried by free ions, from an organic solvent is shown below:^30,31^
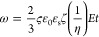
1where ω is the mass of particles deposited, ς is the concentration of particles in solution, ε_0_ and ε_s_ are the relative permittivity of the vacuum and solvent, respectively, ζ is the zeta potential of the suspension, η is the viscosity of the solvent, *E* is the electric field strength, and *t* is the deposition time. From the above equation, the amount of deposition has a relationship with several parameters: concentration, surface charge, field strength, nature of the solvent, and deposition time.

The force acting on the CdSe/CdS NRs in the solution, as shown in Figure 1d, is simply *qE⃗*, where *q* is the net charge on the nanorod and *E⃗* is the electric field. Phosphonic acid and amine-capped nanocrystals generally exhibit positive charges.^32,33^ Therefore, for a positively charged nanorod, the electric field will cause the nanorods in the solution to migrate toward the negative electrode. In our experiment, when an external DC electric field is applied to the CdSe/CdS suspension, the NRs will move toward the ITO/ZnO substrate. The desired net charge on the nanorod to obtain vertically aligned assemblies can be controlled by ligand coverage and the size of the nanorods. With the action of both the electrophoretic mobility and the induced dipole along the long axis, the rods are adequately organized into a three-dimensional (3D) vertical alignment close structure.^34^ The thickness of the NR layer can be precisely controlled by the deposition time and voltage, as demonstrated in Figure S3.

**Figure 2:**
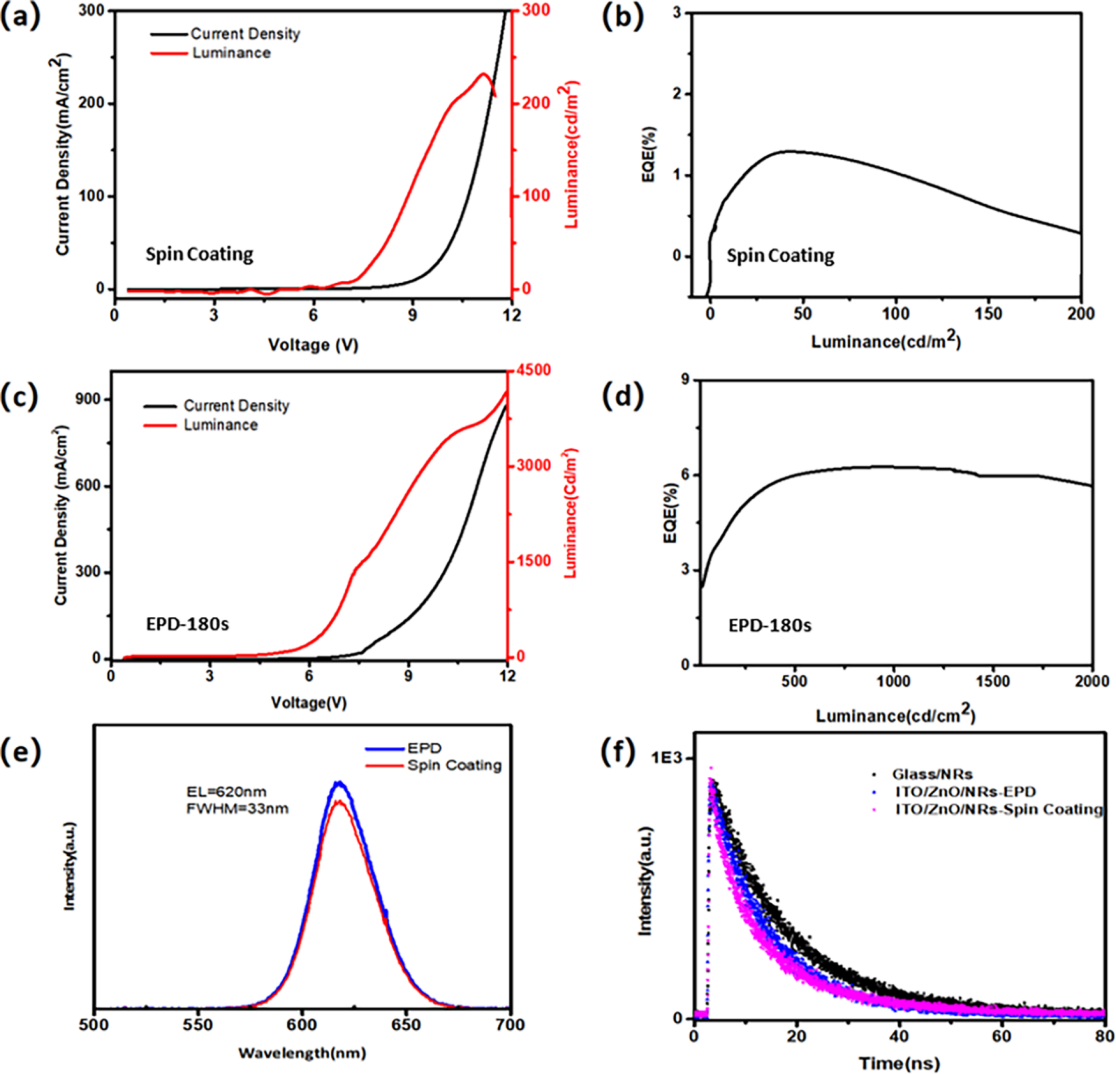
(a)–(d) Current density, luminance versus voltage characteristics, and EQE versus luminance for the NR-LED device by spin-coating (a, b) and EPD (c, d). (e) Normalized EL spectra of devices made by EPD and spin-coating at an applied voltage of 4 V. (f) Time-resolved photoluminescence (TRPL) decay for the NR films. (Device 1 is made by spin-coating; device 2 is made by EPD for 180 s at a field strength of 163 V/cm).

Figure 2a shows the current density–voltage (*J*–*V*) and luminance–voltage (*L*–*V*) characteristics for the spin-coating device (device 1). The turn-on voltage (measured at 1 cd/m^2^), maximum luminance, and maximum EQE NR-LEDs are 5.1 V, 232 cd/m^2^, and 1.32% (Figure 2b), respectively. Compared with the spin-coating LED, we obtain a better performance from the EPD device (device 2): a turn-on voltage of 4.6 V, suggesting an efficient injection of holes and electrons into the nanorod layer at low driving voltages, maximum brightness of over 4300 cd/m^2^ at 11 V, shown in Figure 2c, and a peak EQE of 6.3%, which is achieved at a current density of 360 mA/cm^2^ and a brightness of 1350 cd/m^2^. The current density of the EPD LED (device 2) is higher than that of device 1 at the same applied voltage, and the maximum luminance is enhanced from 232 to 4320 cd/m^2^, which is a 19 times improvement.

The devices with the NR layer made by EPD and spin-coating both exhibit an EL emission of 620 nm with an FWHM of 33 nm (Figure 2e). The EL shows a red shift of 2 nm, and the FWHM also broadens by 11 nm compared to their PL emission peak. This may be due to the quantum confined Stark effect under the presence of an external electric field, which is evident in the QD-LED devices as well.^35^ In addition, when the nanorods are aligned parallel to each other, both radiative and nonradiative decay rates are likely to be modified considerably due to the induced scattering and absorption in their neighbors, which can lead to changes in PL intensity and lifetime.^36^ The average photoluminescence lifetime of the NR films decreased from 17 ns (on glass) to 11.5 and 12.6 ns after NRs made contact with ITO/ZnO films by spin-coating and EPD, respectively (Figure 2f). This may be attributed to the charging of the NRs resulting from the difference in work function between NRs and ITO/ZnO.^27^

**Figure 3:**
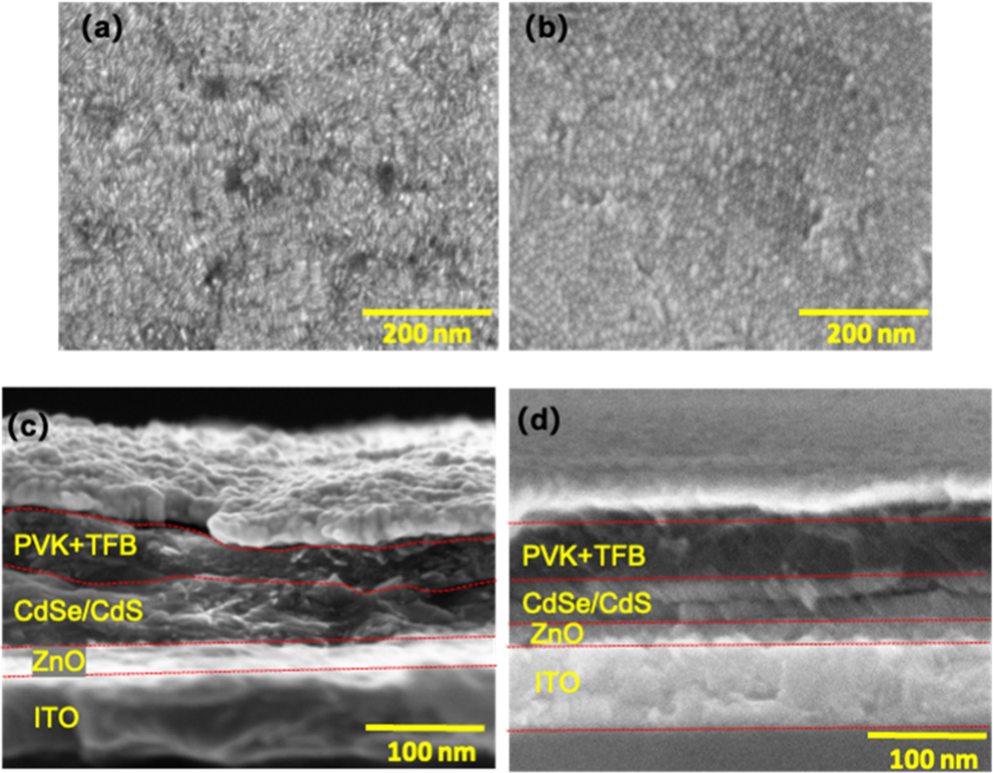
SEM image top view of the NR layer made by spin-coating (a), showing flat-lying NRs, and deposited by EPD (b), showing closely packed honeycomb patterns, on the ZnO layer. Cross-sectional SEM images showing multiple layers of the material with distinct contrast by spin-coating (c) and EPD (d).

The scanning electron microscopy (SEM) images in Figure 3a indicate that the layer deposited by spin-coating is rough, containing many voids, which could lead to direct contact between the HTL and EIL, resulting in transport layer electron–hole recombination. To avoid shorting, a much thicker NR layer needs to be formed (∼50–80 nm in Figure 3c), which may cause nonradiative energy transfer and smaller injection current, leading to low efficiency of the device. The good device efficiency of device 2 can be attributed to the vertical alignment NR layer, which enables better carrier injection and transport within the emission layer. We highlight that the incorporation of the NR layer deposited by EPD for 180 s (device 2, around 55 nm) results in a dense and uniform morphology (Figure 3b) and optimized charge transport in the device. As fewer NR layers are needed for the active film, the electron–hole recombination process within the same NR can happen more effectively. The root-mean-squared roughness of the EPD-NR film is in the range of 5.8–10.6 nm (Figure S2a), which is less than that of the spin-coated device (8.6–15.6 nm, Figure S2b).

The SEM images of the device cross section (ITO/ZnO/NRs/PVK/TFB/Au) are also obtained. The NRs tend to form films of uneven thickness after spin-coating (Figure 3c). As expected, the vertically aligned regions prepared by the EPD method are clearly visible without holes or penetrating cracks (Figure 3d). The thickness of NRs by spin-coating is ∼50 to 80 nm, which is comparable to around 10–16 layers of NRs with 5 nm in diameter and similar to two layers deposited by EPD in length. Based on this assumption, an increase in conductance is expected. The spin-coated device has a larger resistance in comparison to the EPD device. Without efficient radiative recombination to release the energy, excess electron current can deteriorate the NR-LEDs rapidly under operational conditions. Either excess electron injection or overblocking electron current deteriorates the charge balance in the NR-LEDs, thereby degrading the device performance. According to the characterization data, device 2 exhibits an EQE that is 4.7-fold larger than that of the spin-coated device 1, proving that the NRs deposited by EPD effectively improve the device performance. A histogram of peak EQEs obtained from 23 EPD devices with two layers of NRs as the active materials is given as Figure S4 in the Supporting Information.

To explore the effect of the number of layers of the vertically aligned NRs on the device performance, we also fabricated NR-LED devices of different NR thicknesses made by changing the deposition time while keeping the field strength of 163 V/cm in EPD (see Figure S6 for more details). Some characteristics of the devices are summarized in Table 1. The results show that when the thickness of the NR by EPD increases, the current density decreases with the increase in the number of layers. The luminance of the EPD LEDs is optimized at two to three layers. Beyond 3 layers of NRs, the luminance of the devices starts to decrease.

**Table 1:** Summary of the Device Performance of Devices 1–3

device	peak EQE (%)	turn-on voltage	peak luminance (cd/m^2^)	EPD time (s)	thickness (nm)/number of monolayers (ML)
device 1	1.32	5.1	232	spin-coating	50–80/10–16 MLs
device 2	6.3	4.6	4320	180	55/2 MLs
device 3	0.18	5.8	268	60	35/1 ML

Since the CdSe/CdS NRs exhibit a length around 30 nm (Figure S1a), the size of the CdSe/CdS NRs approaches the length scale of electron (hole) mean free path and the conductance within each NR can be regarded as semiballistic transport.^37^ The resistance to charge transport through the film is, therefore, dominated by inter-NR transport. The inter-NR transport is governed by the nearest neighbor hopping at room temperature.^38^ The inter-NR distance is effectively a tunnel barrier, and the rate of carrier hopping is affected by the size of this barrier. To illustrate this effect, we built a 2D finite element model based on drift-diffusion equations (see the Supporting Information for details).

**Figure 4:**
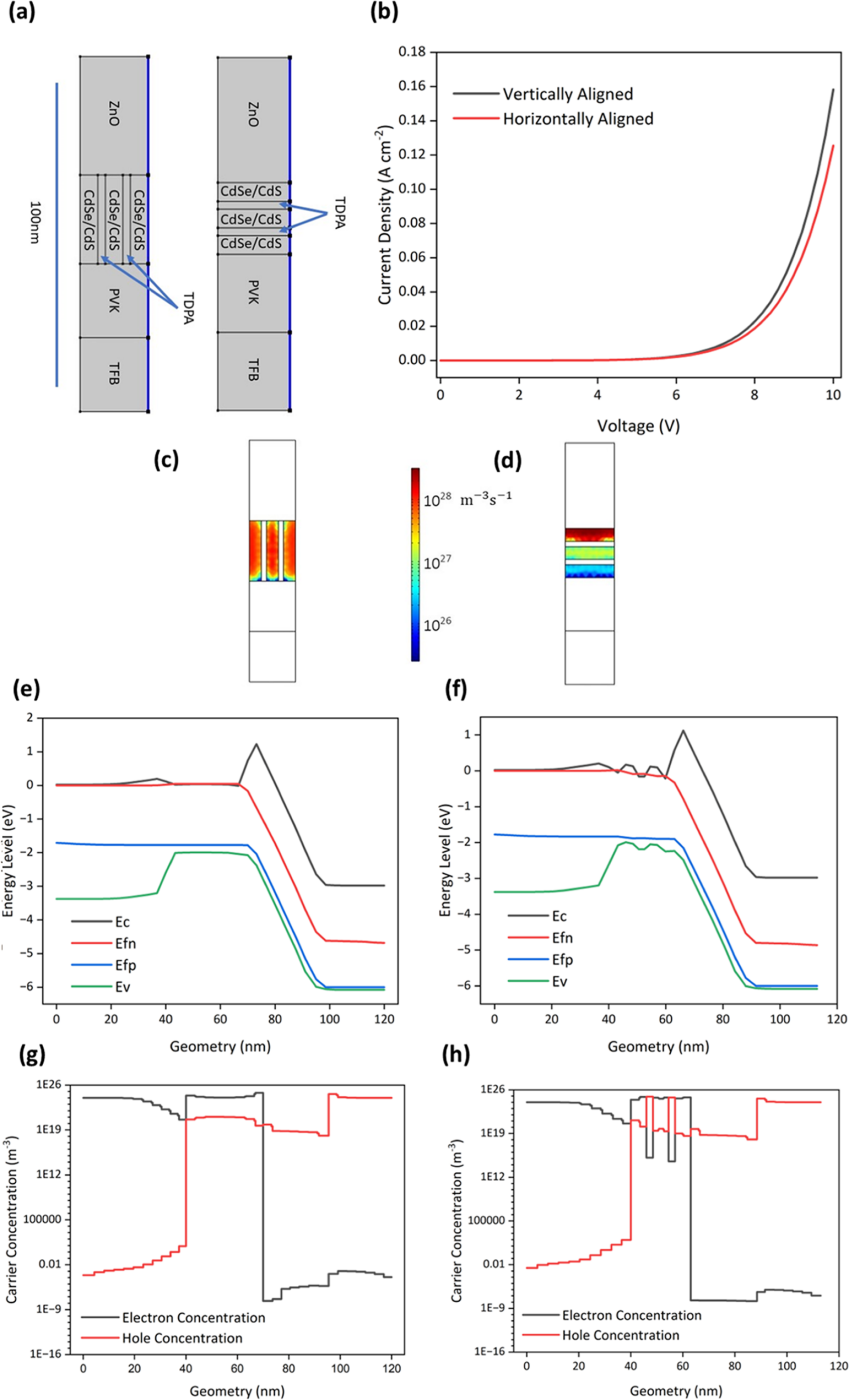
(a) Schematics of the vertically aligned CdSe/CdS NR-LED model and the horizontally aligned CdSe/CdS model with the TDPA ligand defined in COMSOL 6.0. (b) Current density of the NR-LED models from 0 to 10 V for the vertically aligned NR model and the horizontally aligned NR model with tunneling through the ligand allowed. (c, d) Spontaneous emission recombination rate of the NR-LED models in the active region at 6 V for the vertically aligned NR model and the horizontally aligned NR model. (e, f) Energy level diagram of the NR-LED models at 6 V for the vertically aligned NR model and the horizontally aligned NR model. (g, h) Electron and hole concentration throughout the NR-LED models at 6 V for the vertically aligned NR model and the horizontally aligned NR model. The energy levels and carrier concentrations are plotted vs the position outlined by the blue lines in panel (a).

Figure 4a shows the geometries of a model mimicking vertically aligned NRs and another model describing flat-lying NR layers. The geometric parameters of different layers in the vertically aligned NR model are ZnO (40 nm × 23 nm, thickness × width), CdSe/CdS (30 nm × 6 nm, length × width for each NR), TDPA (30 nm × 2.5 nm, thickness × width), PVK (25 nm × 23 nm, thickness × width), and TFB (25 nm × 23 nm, thickness × width), while the parameters of the layers for the horizontally aligned NR model are ZnO (40 nm × 23 nm, thickness × width), CdSe/CdS (6 nm × 23 nm, thickness × width for each layer), TDPA (2.5 nm × 23 nm, thickness × width for each layer), PVK (25 nm × 23 nm, thickness × width), and TFB (25 nm × 23 nm, thickness × width). Figure 4b shows the current density versus voltage from 0 to 10 V for the vertically aligned NR model and the horizontally aligned NR model with tunneling across TDPA layers enabled. The current density is the highest in the case of the vertically aligned NR model as the ligands do not hinder the transport of carriers throughout the active region. Figure 4c,d show the spontaneous emission recombination rates for both the vertically aligned and horizontally aligned NR models at 6 V, respectively. In this case, the maximum spontaneous emission recombination rate is higher in the horizontally aligned NR model than that in the vertically aligned NR model. However, the emission rate integrated over the entire area is higher in the vertically aligned NR model. Figure 4e,f shows the energy level diagrams for both the vertically aligned and horizontally aligned NR models. For the horizontally aligned NR model, when tunneling is enabled, it reduces the potential drop between the three CdSe/CdS layers as the electrons/holes can tunnel through the ligand. Figure 4g,h shows the carrier concentration throughout the device at 6 V for both the vertically aligned and horizontally aligned NR models, respectively. There is a notable drop in the hole concentration across the TDPA ligand layers in the horizontally aligned NR model, which directly impacts the spontaneous emission recombination rate across the three CdSe/CdS layers. More details about the COMSOL simulation can be found in the Supporting Information. This model confirms our experimental findings, where fewer layers of NRs in the LED devices give better device performance. For the experimentally realized vertically aligned monolayer NR-LED case (device 3), it demonstrated the highest current injection. However, it is extremely difficult to eliminate all voids in this case, and we believe that is the reason for the lower EQE in device 3.

## Conclusions

Our work presented NR-based inverted LEDs with the best performance found in vertically aligned CdSe/CdS NRs (2 MLs) as the emitters made by the EPD method, with a luminance of 4320 cd/m^2^ and an EQE of 6.3%. This performance is comparable to the best-reported values for NR-based LEDs in the conventional architecture under a similar voltage.^39^ Such excellent performance is achieved by introducing a conceptually new method for device fabrication resulting from the vertically aligned NR layer between the HTL layer and the oxide EIL. It is found that the vertically aligned NRs can improve the charge balance and reduce the resistance for carrier transport, which would lead to low-cost, large-area, high-efficiency, high-color-quality, stable electroluminescent devices for both display and solid-state lighting technologies.

## Materials and Methods

### Materials

All reagents were used without any further purification. Cadmium oxide (>99%), trioctylphosphine (TOP, 97%), trioctylphosphine oxide (TOPO, 99%), selenium (99.98%), sulfur (99%), tetradecylphosphonic acid (TDPA, >97%), *n*-hexylphosphonic acid (HPA, >99%), poly(9,9-dioctylfluorene-*alt*-*N*-(4-*s*-butylphenyl)-diphenylamine) (TFB, average molecular weight, ∼120,000 g/mol), and poly(9-vinylcarbazole) (PVK) were purchased from Sigma-Aldrich. Patterned ITO-glass substrates (sheet resistance, 20 Ω/sq) were purchased from Xinxiang Ltd.

### Synthesis of CdSe/CdS Nanorods

CdSe quantum dots (QDs) of diameter 3.2 nm were synthesized and purified according to the procedure described by Carbone et al.^22^ with some modifications.

For the CdS shell growth, 0.1158 g of cadmium oxide mixed together with 0.6 g of ODPA, 6 g of TOPO, and 0.162 g of hexylphosphonic acid were mixed in a flask that was then degassed at 150 °C for an hour. The shell growth was started by an injection of 120 μM CdSe QDs dispersed in S/TOP (3.6 mL) at 320 °C in a flask under Ar. The reaction was maintained for 8 min. In the end, the growth was stopped by cooling the reaction down and injecting the 3 mL of toluene at 80 °C. After centrifugation of 5000 rpm with a mixed solution of toluene and ethanol, the core/shell CdSe/CdS nanorod precipitation was dissolved in toluene.

### Preparation of Inverted LED Devices

Glass substrates with patterned indium tin oxide (ITO) were first cleaned with deionized (DI) water, acetone, and isopropanol for 20 min. The substrates were dried by argon. The electron injection layer (EIL), ZnO, was deposited by atomic layer deposition (ALD, Anric Technologies) or electron beam evaporation with a thickness of 40 nm. Next, CdSe/CdS (in toluene, 0.5 mg/mL) was deposited by EPD. For the hole transport layer (HTL), PVK in dioxane (6 mg/mL) and TFB in chloroform (8 mg/mL) were spin-coated layer by layer with 3000 rpm for 30s and annealed at 125 °C for 10 min, respectively. Finally, Au electrodes (40 nm) were deposited using a thermal evaporation system through a shadow mask under a high vacuum of ∼1 × 10^–6^ Torr. The device area was 4 mm^2^ as defined by the overlapping areas of the ITO and Au electrodes. The devices were encapsulated in a glovebox by the cover glasses using ultraviolet-curable resin.

### Electrophoretic Deposition of the CdSe/CdS Nanorod Layer

The as-washed CdSe/CdS nanorods were dispersed in anhydrous toluene as an electrolyte solution and were sonicated for 15 min before deposition. The deposition was undertaken at room temperature, during which the electrodes (two pieces of ITO/ZnO substrate with the size of 30 × 10 mm were attached on both sides, kept at a distance of 2.15 mm, and parallel to each other) were completely immersed in the electrolyte solution with a concentration of 0.5 mg/mL. A 300 V potential was applied for 60–300 s between the two electrodes, with a high-voltage power supply unit (TECHNIX SR-5-F-300) and the voltage was monitored using a digital multimeter (Thurlby Thandar TTi 1604). After deposition, the electrodes were gently raised from the electrolyte solution and dried slowly in the atmosphere slowly. A uniform layer of vertically aligned CdSe/CdS nanorods was observed on the cathode.

### Characterizations

The SEM analysis was undertaken with a Hitachi SU-70 scanning electron microscope. The transmission electron microscopy images of the as-synthesized NRs and device cross-section were characterized using a JEOL JEM-2100F transmission electron microscope (TEM) with 200 keV electron beam energy; the device cross sections were characterized by an FEI Helios G4 CX microscope operated at 5–10 kV. The absorption spectra of the nanocrystals were measured using a Cary Series UV–vis–NIR spectrophotometer. The room temperature PL spectrum of the NRs in toluene was collected by an Ocean Optics 2000+ spectrometer under an excitation wavelength of 405 nm. The absolute photoluminescence quantum yield of the NR film was measured by using an integrating sphere coupled with an Ocean Optics Flame spectrometer.

The current–voltage–luminance characteristics were measured using a Keithley 2602B source, Thorlabs 4P3 integrating sphere, and an HMO3004 oscilloscope coupled to a calibrated PDA200C photodiode amplifier from Thorlabs. The EQE was calculated as the ratio of the photon flux and driving current of the device. The electroluminescence (EL) spectra of the devices were obtained by using an Ocean Optics HR4000+ spectrometer. Time-resolved PL (TRPL) measurements were carried out with a PicoQuant MicroTime 200 STED system, utilizing a 405 nm excitation light source.

## Supporting Information Available

TEM images, XRD, absorption, and PL of CdSe/CdS core–shell NRs; AFM images of morphologies of the CdSe/CdS film made by spin-coating and EPD; influence of field strength and deposition time on the thickness of the CdSe/CdS NR EPD film; histogram of peak EQEs obtained from all EPD devices with two layers of NRs as the active materials; additional SEM images of EPD devices with various NR layer thickness and corresponding device performance and more information on the COMSOL simulation model (PDF)
