# Supplementary material for: Highly Efficient Inverted Light-Emitting Diodes Based on Vertically Aligned CdSe/CdS Nanorod Layers Fabricated by Electrophoretic Deposition
Source: ACS Appl Mater Interfaces. 2024 Feb 15;16(8):10459–67. doi: 10.1021/acsami.3c15542 (PMC10910441; doi:10.1021/acsami.3c15542)
Supplement: Supplementary file 1 — am3c15542_si_001.pdf [file am3c15542_si_001.pdf]

Supporting information

Highly Efficient Inverted Light-Emitting Diodes Based on Vertically Aligned CdSe/CdS Nanorod Layers Fabricated by Electrophoretic Deposition

Yongliang Zhang[1,†], Xuan-Manh Pham[2,†], Thomas Keating[1], Na Jia[2], Anthony Mullen[1], Devika Laishram[1], Mei-Yan Gao[2], Brian Corbett[3], Pai Liu[4*], Xiao Wei Sun[5], Tewfik Soulimane[2], Christophe Silien[1], Kevin M. Ryan[2], Zhenhui Ma[6,*], Ning Liu[1,*]

[1]*Department of Physics and Bernal Institute, University of Limerick, V94 T9PX, Ireland*
[2]*Department of Chemical Sciences and Bernal Institute, University of Limerick, V94 T9PX, Ireland*
[3]*Tyndall National Institute, University College Cork, Cork, T12R5CP, Ireland*
[4]*Shenzhen Key Laboratory of Deep Subwavelength Scale Photonics, Institute of Nanoscience and Applications, Southern University of Science and Technology, Shenzhen 518055, China*
[5]*Institute of Nanoscience and Applications, and Department of Electrical and Electronic Engineering, Southern University of Science and Technology, Nanshan, Shenzhen, Guangdong, 518055, China*
[6]*Department of Physics, Beijing Technology and Business University, Beijing, 100048, China*

Corresponding authors: liup7@sustech.edu.cn, mazh@btbu.edu.cn, ning.liu@ul.ie

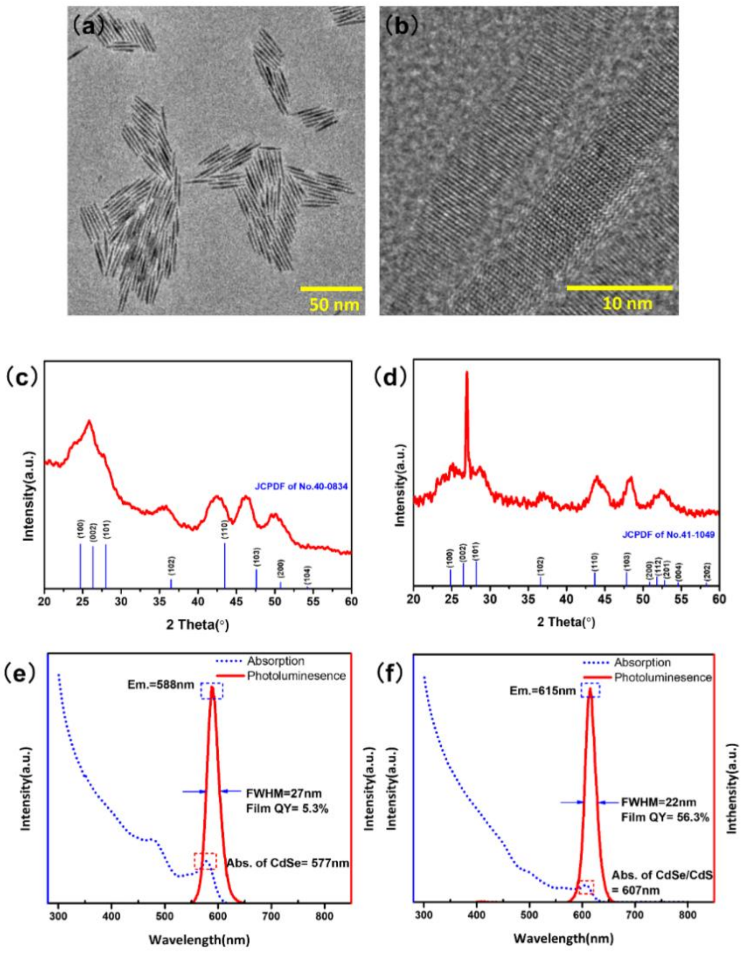

Figure S1 CdSe/CdS core/shell nanorods. (a),(b): A typical TEM image. X-ray diffraction profile of (c) CdSe core and (d) CdSe/CdS core/shell NRs. Ultraviolet–visible absorption and photoluminescence spectra of (e) CdSe core and (f) CdSe/CdS core/shell NRs.

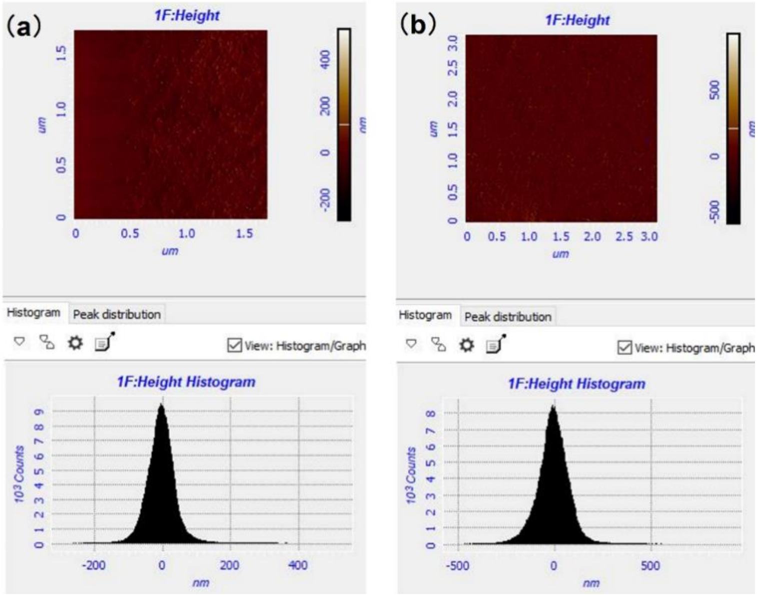

Figure S2 The AFM characteristics for the NRs layers made by (a) spin-coating and (b) EPD.

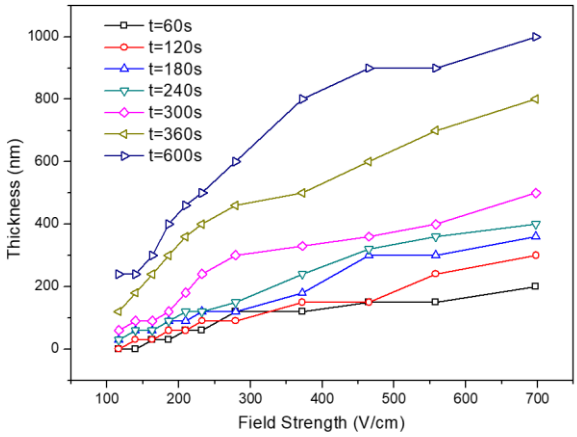

Figure S3 Influence of field strength and deposition time on the thickness of CdSe/CdS NR EPD film. Experimental data points are obtained from EPD of CdSe/CdS NR in Toluene solution with a concentration of 0.5 mg/ml.

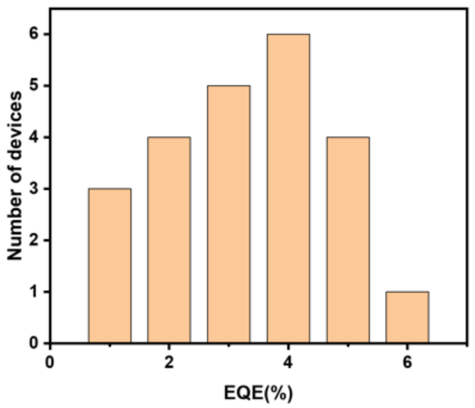

Figure S4 A histogram of peak EQEs obtained from 23 EPD devices with two layers of NR as the active materials.

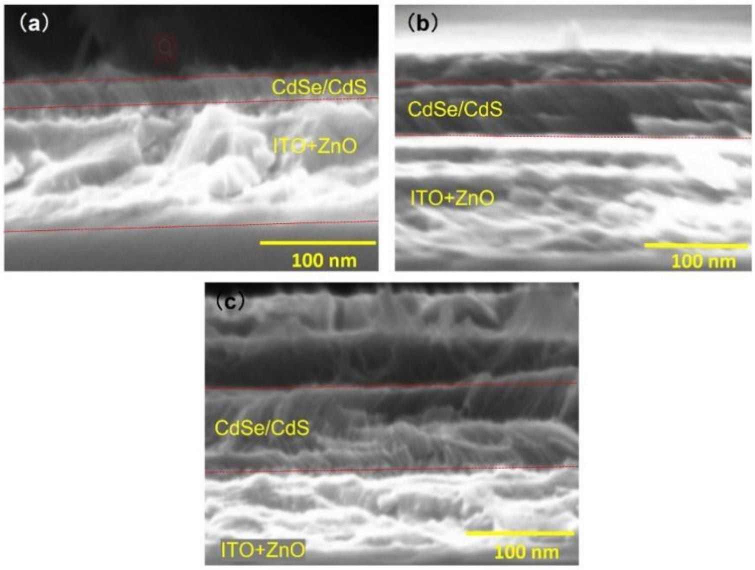

Figure S5 Cross-sectional SEM images showing the NR layers (between the red dash lines) made by EPD with different thickness under 300 V. (a): 60s. (b):210s. (c): 270s.

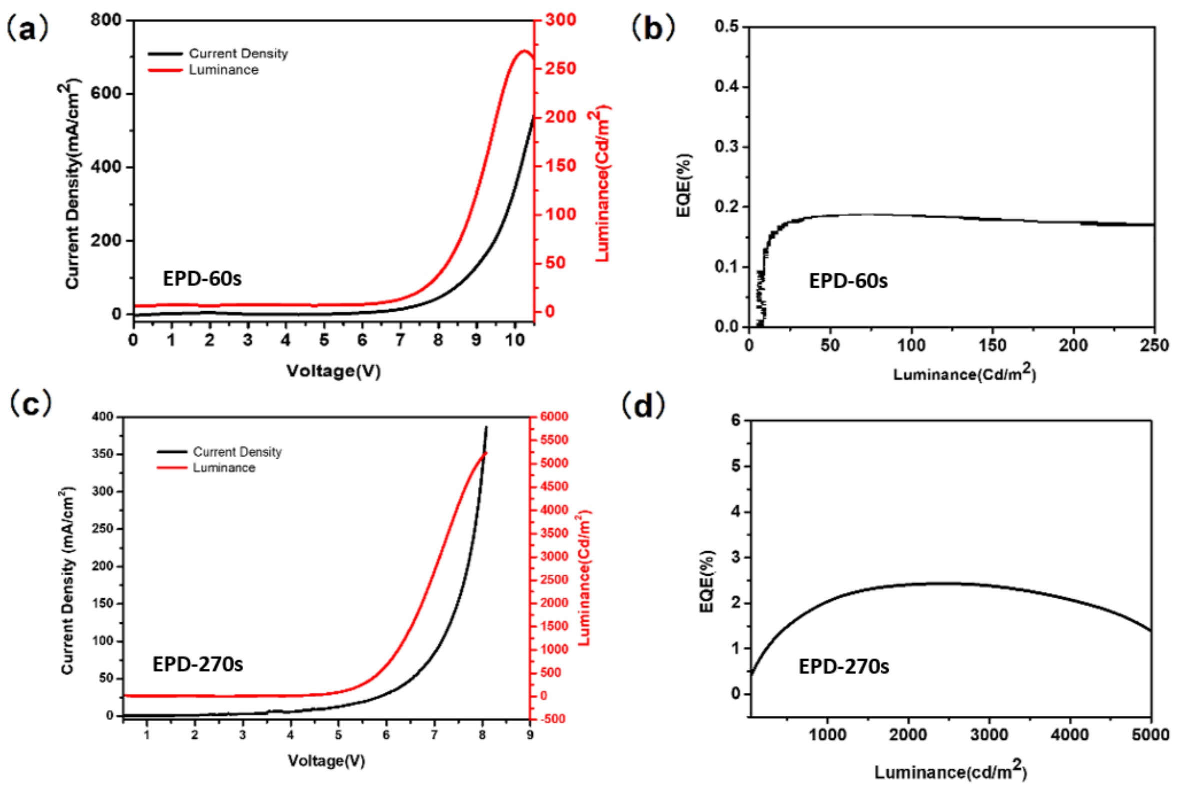

Figure S6 (a)-(d) Current density and luminance versus voltage, and EQE versus luminance characteristics for two more NRs-LEDs by EPD (Device 3: (a) and (b) with deposition time of 60s; Device 4: (c) and (d) with deposition time of 270s).

Please note that the EQE in Device 4 is higher than the spin-coating Device 1 but is lower compared to Device 3. We attribute this to the fine void that might still exist in the macroscale sample. This is supported by the higher current density observed in Device 4 compared to Device 3 at similar voltages.

COMSOL simulations on the 2D model of light emitting diodes

The simulations of the J-V characteristics as well as of the Electron-Hole Concentration plots were conducted by the COMSOL Multiphysics simulation package 6.0 using the semiconductor module. A stationary study was used to calculate the J-V behaviour with and without optical transitions.

Without optical transitions, the semiconductor module in COMSOL 6.0 solves for the drift-diffusion equations of current density $J_n(r)$ ($J_p(r)$) in the defined region:

$$\boldsymbol{J}_n(\boldsymbol{r}) = qn\mu_n \nabla E_c + q\mu_n k_b T G\left(\frac{n}{N_c}\right)\nabla n - qn\mu_n k_b T G\left(\frac{n}{N_c}\right)\nabla ln(N_c) + qnD_{n,th}\nabla ln(T) \quad (1)$$

$$\boldsymbol{J}_p(\boldsymbol{r}) = qp\mu_p \nabla E_v - q\mu_p k_b T G\left(\frac{p}{N_v}\right)\nabla p + qp\mu_p k_b T G\left(\frac{p}{N_v}\right)\nabla ln(N_v) + qpD_{p,th}\nabla ln(T) \quad (2)$$

where n (p) is the electron (hole) density, q is the electron charge, $E_{c(v)}$ is the conduction (valence) band energy level, $\mu_{n(p)}$ is the electron (hole) mobility, k_b is Boltzmann's constant, T is the lattice temperature and $D_{n,\ th(p,\ th)}$ is the thermal diffusion coefficient for electrons (holes). G is the inverse Fermi-Dirac

integral of order 1/2 and $N_{c(v)}$ is the effective density of states for electrons (holes) in the conduction (valence) band energy level.

With optical transitions, current drain due to spontaneous emission needs to be added:

$$0 = \frac{1}{q}(\nabla \cdot \boldsymbol{J}_n) - R_n \quad (3)$$

$$0 = -\frac{1}{q}(\nabla \cdot \boldsymbol{J}_p) - R_p \quad (4)$$

Where $R_{n(p)}$ is the electron (hole) recombination rate that is equal to spontaneous emission rate R_{spon}.

For direct bandgap semiconductors, the R_{spon} is given by:

$$R_{spon} = \int_{E_{g0}}^{\infty} \frac{1}{\tau_{spon}} f_c(1 - f_v) g_{red} dE \quad (5)$$

where g_{red} is the reduced density of states and is defined as:

$$g_{red}(E) = 4\pi \left(\frac{2m_r}{\mathrm{h}^2}\right)^{\frac{3}{2}} \sqrt{\left(E - E_g\right)} \quad (6)$$

where m_r is the reduced mass of CdSe/CdS and τ_{spon} is the spontaneous emission lifetime. h is Planck's constant, c is the speed of light in a vacuum, E is the photon energy that is equal to ħω and E_g is the bandgap of CdSe/CdS. E_{g0} and E_g are the same as no bandgap narrowing was applied to this model. $f_{c(v)}$ is the electron occupancy factors for the conduction (valence) band:

$$f_c = \frac{1}{1+exp\left(\frac{E_{2c}-E_{fn}}{k_bT}\right)} \quad (7)$$

$$f_v = \frac{1}{1+exp\left(\frac{E_{1v}-E_{fp}}{k_bT}\right)} \quad (8)$$

where E_{fn} and E_{fp} are the quasi-Fermi levels of the conduction band and the valence band. E_{2c} and E_{1v} are the energy levels of interest where the difference between the two energy levels is equal to the photon energy, ħω. E_{2c} and E_{1v} are described by equations below:

$$E_{2c} = E_c + \frac{m_r}{m_e}\left(\hbar\omega - E_g\right) \quad (9)$$

$$E_{1v} = E_v - \frac{m_r}{m_h}\left(\hbar\omega - E_g\right) \quad (10)$$

$$\frac{1}{m_r} = \frac{1}{m_e} + \frac{1}{m_h} \quad (11)$$

where E_g is the bandgap of CdSe/CdS, $E_{c(v)}$ is the conduction (valence) band, $m_{e(h)}$ is the effective electron (hole) mass [1].

The effective density of states for the conduction (valence) band, $N_{c(v)}$, was determined from the effective electron (hole) mass, $m_{e(h)}$. The equation for the effective density of states for the conduction and valence bands can be seen below:

$$N_c = 2\left(\frac{2\pi m_e m_0 k_b T}{h^2}\right)^{\frac{3}{2}} \quad (12)$$

$$N_v = 2\left(\frac{2\pi m_h m_0 k_b T}{h^2}\right)^{\frac{3}{2}} \quad (13)$$

where m_0 is the rest mass of the electron.

For the model with the TDPA ligand between three CdSe/CdS nanorods, the WKB tunnelling model for electrons was implemented. The equations for the WKB tunnelling model are as follows:

$$E_{b1} = E_c \tag{14}$$

$$E_{b2} = E_c \tag{15}$$

$$V_{min} = \frac{max(E_{b1}, E_{b2})}{q} \tag{16}$$

$$V_{max} = \frac{\max{(E_c)}}{q} \tag{17}$$

$$\delta = \frac{q}{k_bT} e^{\frac{qV_{max}}{k_bT}} \int_{V_{min}}^{V_{max}} e^{-\frac{qV_x}{k_bT}} e^{-\frac{4\pi}{h}C}\, dV_x \tag{18}$$

$$C = \int_1^2 \sqrt{\max\left(0, 2m(E_b - qV_x)\right)}\, dl \tag{19}$$

Where E_{b1} and E_{b2} are values of E_b, the potential barrier variable, at the two opposite boundaries across the potential barrier. The max function is evaluated in the bounds of the potential barrier domain. δ_{nJn} is the extra current factor which is determined by a double integration along the electrical field line, dl, as well as along the energy axis, dV_x. The integral $\int_1^2 dl$ is a line integral along the electric field line between the two opposite boundaries 1 and 2 across the potential barrier domain.

For ALD or evaporated ZnO films, the relative permittivity was set to 4.17 [2]. The bandgap was set to 3.4 eV and the electron affinity was set to 4 eV [3]. The electron mobility was set to 25 $cm^2\ V^{-1}\ s^{-1}$ and the hole mobility was set to 2.5 $cm^2\ V^{-1}\ s^{-1}$ [4]. The effective electron mass was set to 0.24 and the effective hole mass was set to 0.59 [5]. The donor concentration was set to 1 x 10^{23} m^{-3} and the background doping was set to 1 x 10^{18} m^{-3}. For CdSe/CdS, the relative permittivity was set to 5.8 [6]. The bandgap was set to 2.03 eV and the electron affinity was set to 4.27 eV [3]. The electron mobility was set to 720 $cm^2\ V^{-1}\ s^{-1}$ and the hole mobility was set to 75 $cm^2\ V^{-1}\ s^{-1}$ [7]. The effective electron mass was set to 0.13 and the effective hole mass was set to 0.45 [8]. The spontaneous lifetime of CdSe/CdS was set to 10ns in the optical transitions section [9]. The donor concentration was set to 1 x 10^{24} m^{-3} and the background doping was set to 1 x 10^{18} m^{-3}. For PVK, the permittivity was set to 2.82 [10]. The bandgap was set to 3.6 eV [11]. The electron affinity was set to 2.3 eV [12]. The electron mobility was set to 1 x 10^{-6} $cm^2\ V^{-1}\ s^{-1}$ and the hole mobility was set to 1 x 10^{-7} $cm^2\ V^{-1}\ s^{-1}$ [13]. The effective electron and hole mass were set to 0.55 [14]. For TFB, the permittivity was set to 2.72 [15]. The bandgap was set to 3.1eV and the electron affinity was set to 2.3eV [16]. The electron mobility was set to 2 x 10^{-6} $cm^2\ V^{-1}\ s^{-1}$ and the hole mobility was set to 2 x 10^{-7} $cm^2\ V^{-1}\ s^{-1}$ [17]. The effective electron and hole mass were set to 1. The acceptor concentration was set to 1 x 10^{24} m^{-3} and the background doping was set to 1 x 10^{20} m^{-3}. The ligand used was TDPA. The relative permittivity was set to 2.14. The bandgap was set to 2.3 eV and the electron affinity was set to 3.6 eV. Both the electron and hole mobility values and the effective electron and hole mass values were set to be the same as the mobility values of CdSe/CdS. The acceptor concentration was set to 1 x 10^{24} m^{-3} and the background doping was set to 1 x 10^{20} m^{-3}.

CdSe/CdS Vertically Aligned NR model

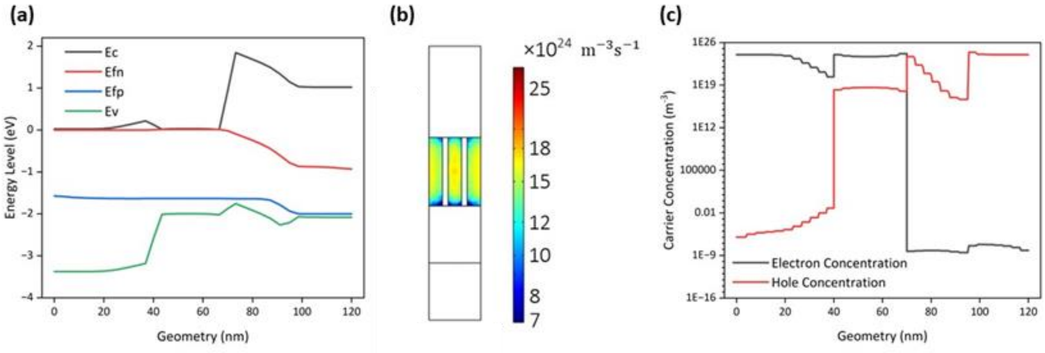

Figure S7 (a) Energy level diagram of the vertically aligned NR-LED model at 2V. The geometric parameters are the same as used in Figure 4. The black curve corresponds to the conduction band edge, E_c, and the green curve corresponds to the valence band edge, E_v. The red curve represents the quasi-fermi energy level for electrons, E_{fn}, and the blue curve corresponds to the quasi-fermi energy level for holes, E_{fp}. (b) Spontaneous emission recombination rate of the NR-LED in the active region at 2V. (c) Electron-Hole concentration throughout the NR-LED model at 2V.

At 2V, the quasi-fermi level for electrons is close to or above the energy level of the conduction band edge in the CdSe/CdS region and the quasi-fermi level for holes is closer to the valence band edge meaning the valence band will be filled with holes. The emission rate is uniform throughout the active region.

CdSe/CdS horizontally aligned model with tunnelling

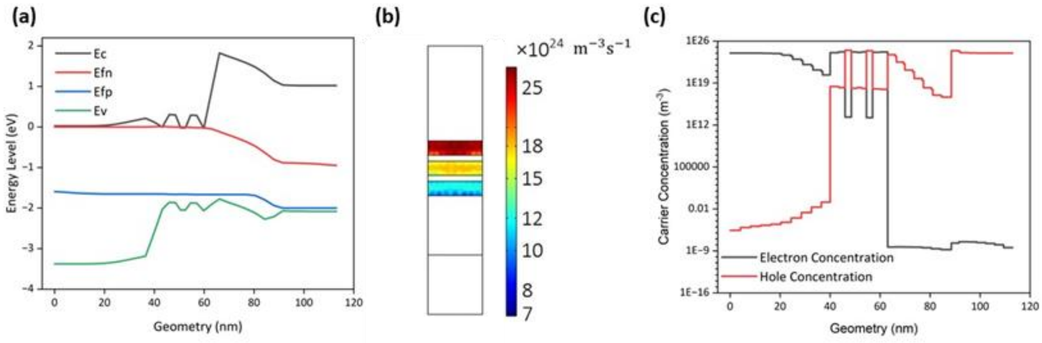

Figure S8 (a) Energy level diagram of the NR-LED model at 2V for the horizontally aligned model with tunnelling between the two adjacent CdSe/CdS layers across the TDPA. The geometric parameters are the same as used in Figure 4. (b) Spontaneous emission recombination rate of the NR-LED in the active region and (c) the corresponding Electron-Hole concentration throughout the NR-LED model at 2V.

At 2V, the quasi-fermi level for electrons is close to or above the energy level of the conduction band edge in the CdSe/CdS region and the quasi-fermi level for holes is closer to the valence band edge meaning the valence band will be filled with holes. With tunnelling enabled, the potential drop is less notable as both electrons and holes can tunnel through the ligand. Please note that the carriers tunnelling through the TDPA layers is required for the simulation to converge.

References:

[1] *Semiconductor Module User's Guide, COMSOL 5.4* (2018).

[2] *A. Jazmati, B. Abdullah, "Optical and Structural Study of ZnO Thin Films Deposited by RF Magnetron Sputtering at Different Thicknesses: A Comparison with Single Crystal", Mat. Res. 21, 3* (2018).

[3] *L. Tzeng, C. Cheng, and Y. Chen, "Enhancement of band-edge emission induced by defect transition in the composite of ZnO nanorods and CdSe/ZnS quantum dots, L. Tzeng, C. Cheng, and Y. Chen", Optics Letters, Vol. 33, No. 6* (2008).

[4] *V. Nguyen, D. Bellet, B. Masenelli and D. Muñoz-Rojas, "Increasing the Electron Mobility of ZnO-Based Transparent Conductive Films Deposited by Open-Air Methods for Enhanced Sensing Performance", ACS Appl. Nano Mater, 1, 6922-6931* (2018).

[5] *V. Sirkeli, O. Yilmazoglu, F. Küppers and H. Hartnagel, "Effect of p-NiO and n-ZnSe interlayers on the efficiency of p-GaN/n-ZnO light-emitting diode structures", Semiconductor Science and Technology, 30, 6, 065005* (2015).

[6] *T. Wakaoka et al, "Confined synthesis of CdSe quantum dots in the pores of metal–organic frameworks", Journals of Materials Chemistry, 2, 7173-7175* (2014).

[7] *C. Canali, F. Nava and G. Ottaviani, "Hole and Electron Drift Velocity in CdSe at Room Temperature", Solid State Communications, Vol.11, pp. 105-107* (1972).

[8] *S. Lohmann et al, "Influence of Interface-Driven Strain on the Spectral Diffusion Properties of Core/Shell CdSe/CdS Dot/Rod Nanoparticles", J. Phys. Chem. C, 123, 5099-5109* (2019).

[9] *I. Coropceanu et al, "Slow-Injection Growth of Seeded CdSe/CdS Nanorods with Unity Fluorescence Quantum Yield and Complete Shell to Core Energy Transfer", ACS Nano, 10, 3295-3301* (2016).

[10] *A. Ritchie et al, "Tunable High Refractive Index Polymer Hybrid and Polymer-Inorganic Nanocomposite Coatings", ACS Applied Materials & Interfaces, 13, 28, 33477-33484 (2021).*

[11] *M. Azadinia et al, "Improved performance of photoconductive gain hybrid UV detector by trap state engineering of ZnO nanoparticles", Journal of Applied Physics, 122, 154501 (2017).*

[12] *Y. Liu et al, "Highly Efficient All-Solution Processed Inverted Quantum Dots Based Light Emitting Diodes", ACS Nano, 12, 1564-1570 (2018).*

[13] *D. Pai, J Yanus & M. Stolka, "Trap-Controlled Hopping Transport", J. Phys. Chem., 88, 4714-4717 (1984).*

[14] *P. D'Angelo et al, "Electrical transport properties characterization of PVK (poly N-vinyl carbazole) for electroluminescent devices applications", Solid-State Electronics, 51, 123-129 (2007).*

[15] *G. Mei et al, "Optical Tunneling to Improve Light Extraction in Quantum Dot and Perovskite Light-Emitting Diodes", IEEE Photonics Journal, 12, 6, 1-14 (2020).*

[16] *N. Kumawat, W. Tress & F. Gao, "Mobile ions determine the luminescence yield of perovskite light-emitting diodes under pulsed operation", Nature Communications, 12, 4899 (2021).*

[17] *J. Chen et al, "Highly efficient all-solution processed blue quantum dot light-emitting diodes based on balanced charge injection achieved by double hole transport layers", Organic Electronics, 94 (2021).*
